# Performance of noninvasive ventilation in acute respiratory failure in critically ill patients: a prospective, observational, cohort study

**DOI:** 10.1186/s12890-015-0139-3

**Published:** 2015-11-11

**Authors:** Thiago Domingos Corrêa, Paula Rodrigues Sanches, Lúbia Caus de Morais, Farah Christina Scarin, Eliézer Silva, Carmen Sílvia Valente Barbas

**Affiliations:** Intensive Care Unit, Hospital Israelita Albert Einstein, Av. Albert Einstein, 627/701, 5° andar, São Paulo, CEP: 05651-901 Brazil; Pulmonary and Critical Care Division- INCOR, University of São Paulo, São Paulo, Brazil

**Keywords:** Respiratory insufficiency, Noninvasive ventilation, Hypoxemia, Intensive care unit, Mortality, Outcomes

## Abstract

**Background:**

Noninvasive ventilation (NIV) is used in critically ill patients with acute respiratory failure (ARF) to avoid endotracheal intubation. However, the impact of NIV use on ARF patient’s outcomes is still unclear. Our objectives were to evaluate the rate of NIV failure in hypoxemic patients with an arterial carbon dioxide partial pressure (PaCO_2_) < 45 mmHg or ≥ 45 mmHg at ICU admission, the predictors of NIV failure, ICU and hospital length of stay and 28-day mortality.

**Methods:**

Prospective single center cohort study. All consecutive patients admitted to a mixed ICU during a three-month period who received NIV, except for palliative care purposes, were included in this study. Demographic data, APACHE II score, cause of ARF, number of patients that received NIV, incidence of NIV failure, length of ICU, hospital stay and mortality rate were compared between NIV failure and success groups.

**Results:**

Eighty-five from 462 patients (18.4 %) received NIV and 26/85 (30.6 %) required invasive mechanical ventilation. NIV failure patients were comparatively younger (67 ± 21 vs. 77 ± 14 years; *p* = 0.031), had lower arterial bicarbonate (*p* = 0.005), lower PaCO_2_ levels (*p* = 0.032), higher arterial lactate levels (*p* = 0.046) and APACHE II score (*p* = 0.034) compared to NIV success patients. NIV failure occurred in 25.0 % of patients with PaCO_2_ ≥ 45 mmHg and in 33.3 % of patients with PaCO_2_ < 45 mmHg (*p* = 0.435). NIV failure was associated with an increased risk of in-hospital death (OR 4.64, 95 % CI 1.52 to 14.18; *p* = 0.007) and length [median (IQR)] of ICU [12 days (8–31) vs. 2 days (1–4); *p* < 0.001] and hospital [30 (19–42) vs. 15 (9–33) days; *p* = 0.010] stay. Predictors of NIV failure included age (OR 0.96, 95 % CI 0.93 to 0.99; *p* = 0.007) and APACHE II score (OR 1.13, 95 % CI 1.02 to 1.25; *p* = 0.018).

**Conclusion:**

NIV failure was associated with an increased risk of in-hospital death, ICU and hospital stay and was not affected by baseline PaCO_2_ levels. Patients that failed were comparatively younger and had higher APACHE II score, suggesting the need for a careful selection of patients that might benefit from NIV. A well-designed study on the impact of a short monitored NIV trial on outcomes is needed.

## Background

Noninvasive ventilation (NIV) has been established as a useful and safe method to improve gas exchange for critically ill patients with different etiologies of acute respiratory failure (ARF) [1, 2]. NIV decreases work of breathing, improves arterial oxygenation and alveolar ventilation, prevents the use of invasive mechanical ventilation, reduces the incidence of ventilator associated pneumonia, decreases the length of intensive care unit (ICU) stay and mortality mainly due to chronic obstructive pulmonary disease exacerbations [3, 4] and acute cardiogenic pulmonary edema [5–8].

Nevertheless, the use of NIV to support other etiologies of ARF remains controversial [9–11]. The multifactorial etiology and the heterogeneity of patients classified as ARF patients may justify different results obtained with NIV application [12]. The available evidence suggests caution in the use of NIV in patients with acute hypoxemic respiratory failure especially in acute respiratory distress syndrome (ARDS) and community-acquired pneumonia due to high NIV failure rates [11, 13, 14].

The overall incidence of NIV failure defined by the need of intubation and invasive mechanical ventilation reported in the literature can vary widely, approaching 50 % in patients with community-acquired pneumonia and ARDS [11, 15]. The reasons for NIV failure are most commonly related to the incapacity to improve oxygenation, inability to correct dyspnea, incapacity to manage copious secretions, mask discomfort, agitation, anxiety, hemodynamic instability and progression of ARF [15]. Delayed identification of patients who fail on NIV may result in late intubation and initiation of invasive mechanical ventilation, which have been associated with increased morbidity and mortality [11].

Therefore, it is imperative to identify the variables that can help predict patients who will fail on NIV as early as possible, and thus allow a prompt intubation in cases it will be necessary [11].

Our objective was to evaluate the rate of NIV failure in hypoxemic patients with an arterial carbon dioxide partial pressure (PaCO_2_) lower than 45 mmHg or equal to or higher than 45 mmHg at ICU admission. We also aimed to evaluate the predictors of NIV failure, intensive care and hospital length of stay, mortality rate at day 28 and the main complications associated with NIV.

## Methods

### Study design and patient selection

This prospective observational single center cohort study was conducted in a forty-one bed, open mixed ICU of a tertiary care hospital in São Paulo, Brazil. This study was approved by the institutional review board of Hospital Albert Einstein, who waived the need for informed consent in view of the observational characteristic of the study (protocol number: 19301213.5.0000.0071).

During a three-month period, all consecutive patients admitted to the ICU that presented a peripheral oxygen saturation (SpO_2_) lower than 90 % despite oxygen delivered through a Venturi Mask [fraction of inspired oxygen (FiO_2_) around of 50 %] or by an oxygen bag (FiO_2_ around 100 %) that received NIV, except for palliative care purposes, were included in this study [16].

Patients were excluded when they were under eighteen, had previous tracheostomy, used NIV for palliative care or presented contraindications to receiving NIV, including cardiac or respiratory arrest, Glasgow Coma Scale ≤ 10, severe upper gastrointestinal bleeding, hemodynamic instability, unstable cardiac arrhythmia, facial surgery or trauma, upper airway obstruction, inability to cooperate or protect the airway, inability to clear respiratory secretions or high risk for aspiration. The researches followed the patients and did not interfere in the ICU medical and multidisciplinary staff decisions.

### Protocol of niv use in the ICU

Noninvasive ventilation was applied to patients admitted to the ICU that presented a SpO_2_ lower than 90 % despite oxygen delivered through a Venturi Mask (FiO_2_ around of 50 %) or by an oxygen bag (FiO_2_ around 100 %) [16]. Noninvasive ventilation was delivered by a total face mask, secured with head straps, coupled to a BIPAP Vision™ (Respironics INC®, Pennsylvania, USA). For patients with a nasogastric tube, a seal connector in the dome of the mask was used to minimize air leakage. After the mask was attached to the patient, pressure support could be increased from 5 up to 20 cm H_2_O to obtain an exhaled tidal volume of 6 mL/kg of predicted body weight, a respiratory rate lower than 30 breaths per minute, attenuation of respiratory accessory muscle activity and achievement of patient’s comfort. Positive end-expiratory pressure (PEEP) was initiated at 5 cm H_2_O and increased in steps of 2 to 3 cm H_2_O up to 15 cm H_2_O until the FiO_2_ requirement was 60 % or less in patients with hypoxemic respiratory failure.

All ventilator settings could be re-adjusted by the attending physician and by a chest physiotherapist, based on the results of continuous oximetry, measurements of arterial blood gases (specially PaCO_2_ and pH) and ventilator parameters (expiratory tidal volume, respiratory rate, and mask leakage) as well as on patients’ comfort. A baseline arterial blood gas analysis was performed after patient’s stabilization on NIV.

Patients did not usually receive sedatives. If they were agitated and uncomfortable with the mask, intravenous morphine or dexmedetomidine was initiated [17]. All patients were monitored with continuous electrocardiography and SpO_2_. The heads of the beds were kept elevated at 30°. Each patient was evaluated periodically according to the institutional protocol by the attending physician and by a respiratory physiotherapist in order to access the possibility to reduce or increase PEEP or NIV discontinuation/continuation.

NIV success patients were maintained coupled to a BIPAP vision continuously during a 24-h period. Afterwards, NIV parameters were re-adjusted based on SpO_2_, arterial blood gas analysis (specially PaCO_2_ levels), ventilator parameters (expiratory tidal volume, respiratory rate and mask leakage) and patient’s comfort. When FiO_2_ was lower than 50 %, respiratory rate lower than 30 breaths per minute, expiratory tidal volume higher than 5 mL/kg of predicted body weight with a pressure support lower than 10 cm H_2_O and PEEP lower than 8 cm H_2_O, NIV was discontinued and oxygen ventury mask of 50 % initiated. If an oxygen ventury mask of 50 % was well tolerated during a one-hour period, the ventury mask of 50 % was alternated with NIV (1 h in ventury mask of 50 % and 3 h in NIV) until the patient could stay spontaneously breathing. The maximal time allowed on full NIV support was 24 h. After 24 h on NIV, patients that could not stay for at least one hour on oxygen ventury mask was defined dependent on NIV and was intubated and mechanically ventilated.

### Endotracheal intubation

Detection of NIV failure, the decision to intubate patients and start mechanical ventilation were made by the attending physician. Patients who failed treatment with NIV underwent endotracheal intubation with cuffed endotracheal tubes (internal diameter of 7.5 to 8.5 mm) and were mechanically ventilated (Servo-i; Maquet Critical Care, Solna, Sweden).

Criteria for endotracheal intubation included failure to maintain an arterial oxygen partial pressure (PaO_2_) > 60 mmHg or SpO_2_ > 90 % with an FiO_2_ equal to or greater than 60 %, PaCO_2_ higher than 60 mmHg with pH lower than 7.25, inability to protect the airways or to manage copious tracheal secretions, hemodynamic or electrocardiographic instability, inability to tolerate the face mask, inability to correct dyspnea and progression of respiratory failure [16].

### Outcome measures

Demographic data, etiology of respiratory failure, APACHE II score [18], vital signs, electrolytes, hemoglobin, platelets, white blood cell count, serum creatinine, arterial lactate, FiO_2_, ratio of the arterial oxygen partial pressure to the fraction of inspired oxygen (PaO_2_/FiO_2_), arterial pH, PaCO_2_, arterial lactate, number of patients that used NIV, number of patients that needed endotracheal intubation (NIV failure), in-hospital mortality rate, mortality at day 28, length of ICU and hospital stay and complications related to NIV were recorded.

Our primary outcome was the incidence of NIV failure, defined by the need of endotracheal intubation and mechanical ventilation in hypoxemic patients with PaCO_2_ < 45 mmHg and ≥ 45 mmHg at ICU admission. Secondary outcomes were the main indications for acute application of NIV, the predictors of NIV failure, ICU and hospital lengths of stay, in-hospital and mortality at day 28 and the main complications associated with noninvasive ventilation.

### Statistical analysis

Categorical variables were displayed as absolute and relative frequencies. Numerical variables were presented as mean and standard deviation (SD) or median with interquartile ranges (IQR) in case of non-normal distribution, tested by the Kolmogorov-Smirnov test.

Comparisons were made between NIV failure and NIV success groups and between patients with PaCO_2_ < 45 mmHg and ≥ 45 mmHg at ICU admission. Categorical variables were compared with chi-square test or with Fisher exact test when appropriate. Continuous variables were compared using independent *t* test or Mann–Whitney *U* test in case of non-normal distribution. Survival curves at day 28 were performed according to the Kaplan-Meier method and compared with a log-rank test.

A univariate logistic regression analysis was performed to identify which factors (predictors) were associated with NIV failure. Only variables presented in more than five patients in each group were included. A multivariate logistic regression analysis with backward elimination procedure including all predictors showing a *p* value ≤ 0.25 in the univariate analysis was undertaken to obtain an adjusted odds ratio (OR) with 95 % confidence interval (CI) and define which variables were independently associated with NIV failure.

Statistical tests were 2-sided, and a *p* < 0.05 was considered statistically significant. Statistical analyses were performed using IBM® SPSS® Statistics version 22.0 for Windows.

## Results

### Patients

**Fig. 1:**
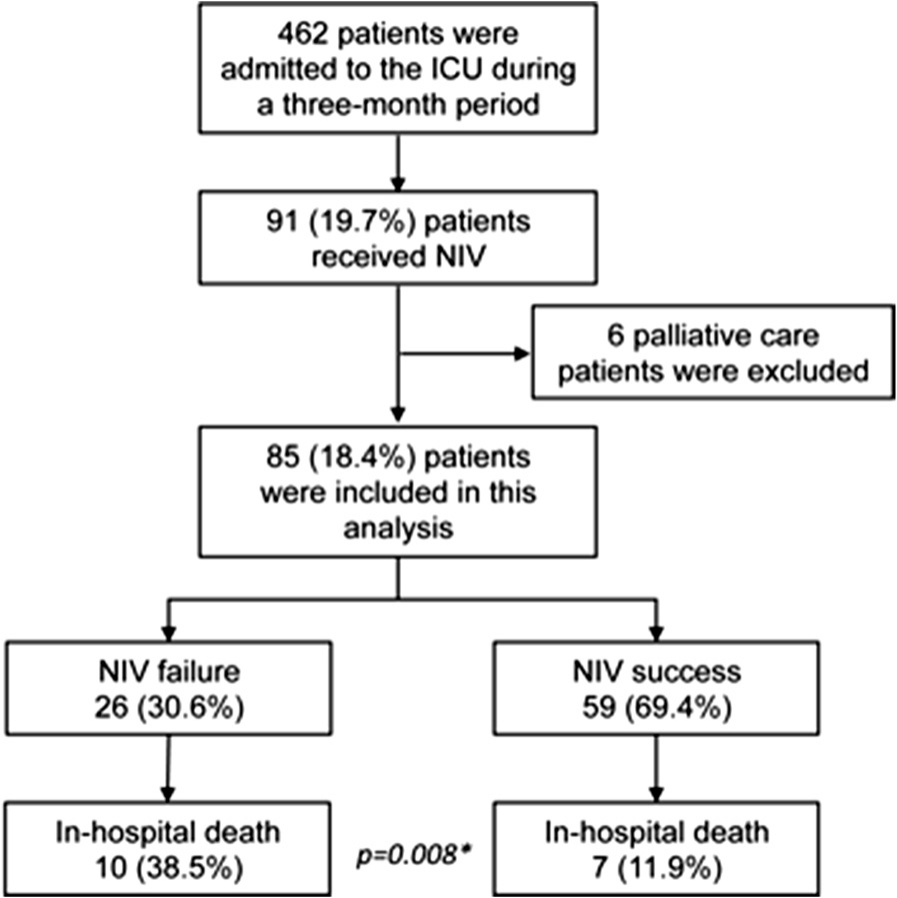
Study flow diagram. NIV = noninvasive ventilation, * = *p* value comparing in-hospital mortality between NIV failure vs. NIV success Groups

In a three-month period, 462 patients were admitted to the ICU. Ninety-one patients fulfilled the criteria for NIV use, but six patients were excluded because they used NIV for palliative care purposes. Therefore, eighty-five patients were included in the study (Fig. 1).

The baseline characteristics, clinical, physiological and laboratorial parameters of studied patients are presented on Table 1. NIV failure patients were comparatively younger, had lower arterial bicarbonate, and lower PaCO_2_ levels and had higher arterial lactate levels and APACHE II score compared to NIV success patients (Table 1). The main etiologies of acute respiratory failure did not differ between the two groups (Table 2). Concerning comorbidities, NIV failure group had a higher number of transplanted patients in comparison to NIV success group (Table 1).

**Table 1:** Baseline characteristics of study patients

Characteristics	NIV failure 26 (30.6 %)	NIV success 59 (69.4 %)	*P* value^**¶**^
Age (years)	67 (21)	77 (14)	0.031^a^
Male gender, n° (%)	17 (65.4)	27 (45.8)	0.106^b^
Mean arterial pressure (mmHg)	81 (24)	93 (21)	0.032^a^
Heart rate (beats/min)	107 (25)	95 (19)	0.031^a^
APACHE II score	16.0 (4.7)	13.4 (5.3)	0.034^a^
Arterial pH	7.36 (0.08)	7.39 (0.07)	0.106^a^
Bicarbonate (mmol/L)	19.6 (7.3)	23.7 (4.7)	0.005^a^
PaCO_2_ (mmHg), median [IQR]	30.8 [26.6–40.1]	35.3 [31.5–43.5]	0.032^c^
PaCO_2_ ≥ 45 mmHg, n° (%)	7 (26.9)	21 (35.6)	0.466^b^
PaO_2_/FiO_2_	277 (148)	282 (109)	0.878^a^
Arterial lactate (mg/dl), median [IQR]	17 [14–26]	12 [8–21]	0.046^c^
Hemoglobin (mg/dl)	10.6 (2.1)	11.0 (2.1)	0.499^a^
Reason for ICU admission, n° (%)			
Medical	20 (76.9)	53 (89.8)	0.174^b^
Operative	6 (23.1)	6 (10.2)	
Comorbidities, n (%)			
Diabetes Mellitus	9 (34.6)	15 (25.4)	0.438^b^
Transplantation	7 (26.9)	2 (3.4)	0.003^b^
Chronic renal failure	6 (23.1)	8 (13.6)	0.344^b^
Systemic hypertension	6 (23.1)	17 (28.8)	0.792^b^
COPD	6 (23.1)	12 (20.3)	0.779^b^
Liver cirrhosis	4 (15.4)	1 (1.7)	0.029^b^
Coronary insufficiency	4 (15.4)	12 (20.3)	0.766^b^
Congestive heart failure	4 (15.4)	22 (37.3)	0.072^b^
Neoplasm	3 (11.5)	7 (11.9)	1.000^b^
None	2 (7.7)	12 (20.3)	0.209^b^

Values are mean (SD) or median [IQR] when indicated

¶ = *p* values and the respective statistical tests comparing NIV failure vs. NIV success groups. ^a^ = Independent *t*-test, ^b^ = Fisher’s exact test, ^c^ = Mann–Whitney *U* test. *APACHE* II Acute physiology and chronic health evaluation II (The score can range from 0 to 71, with higher scores indicating more severe illness), *PaCO*_*2*_ Partial pressure of arterial carbon dioxide, and *PaO*_*2*_ */FiO*_*2*_ Ratio of the arterial oxygen partial pressure to the fraction of inspired oxygen, *ICU* Intensive care unit, *COPD* Chronic obstructive pulmonary disease

**Table 2:** Main causes of acute respiratory failure

Causes of failure, n (%)	NIV failure 26 (20.6 %)	NIV success 59 (69.4 %)	*P* value^¶^
Community acquired pneumonia	10 (38.5)	20 (33.9)	0.806
Cardiogenic pulmonary edema	4 (15.4)	15 (25.4)	0.402
Acute respiratory distress syndrome	5 (19.2)	5 (8.5)	0.271
Acute COPD	3 (11.5)	7 (11.9)	1.000
Other causes of ARF^a^	4 (15.4)	12 (20.3)	0.766

¶ = *p* values with Fisher’s exact test comparing NIV failure vs. NIV success groups. *COPD* Chronic obstructive pulmonary disease, ^a^ = mucous plugging, atelectasis, pulmonary embolism, pulmonary contusion and neuromuscular disease

### Response to NIV and complications

NIV success occurred in 69.4 % (59/85) of patients (NIV Success Group) and NIV failure occurred in 30.6 % (26/85) of patients that needed intubation and mechanical ventilation (NIV failure Group) (Table 1 and Fig. 1). NIV failure occurred in 25.0 % (7/28) of patients with PaCO_2_ ≥ 45 mmHg and in 33.3 % (19/57) of patients with PaCO_2_ < 45 mmHg (OR 0.67, 95 % CI 0.24 to 1.84; *p* = 0.435) (Table 1).

In 61.5 % (16/26) of patients, NIV failure occurred during the first 24 h of noninvasive mechanical ventilation. The main reasons for endotracheal intubation included progression of hypoxemia in 65.4 % (17/26), neurological deterioration in 19.2 % (5/26), gastric distension 7.7 % (2/26), hemodynamic instability 3.8 % (1/26) and patients’ dangerous agitation 3.8 % (1/26) (Table 2).

The only complication associated with NIV was gastric distension reported in 3/26 (11.5 %) NIV failure patients vs. 4/59 (6.8 %) in NIV success groups (*p* = 0.670; Table 3).

**Table 3:** Mortality rate, length of stay and incidence of complications associated with noninvasive positive pressure ventilation

Variables	NIV failure 26 (30.6 %)	NIV success 59 (69.4 %)	*P* value^¶^
Length of ICU stay (days)	12 [8–31]	2 [1–4]	<0.001^a^
Length of hospital stay (days)	30 [19–42]	15 [9–33]	0.010^a^
Mortality at day 28, n° (%)	5 (19.2)	4 (6.8)	0.124^b^
In-hospital mortality, n° (%)	10 (38.5)	7 (11.9)	0.008^b^
Complications associated with NIV			
Gastric distension	3 (11.5)	4 (6.8)	0.670^b^

¶ = *p* values for NIV failure vs. NIV success Groups. ^a^ = Mann–Whitney *U* test, ^b^ = Fisher’s exact test and. *ICU* Intensive care unit. Values are median [IQR] or n° (%) when indicated

### Length of ICU and hospital stay

The median lengths of ICU and hospital stays were significantly higher in NIV failure in comparison to the NIV success groups (Table 3). The median (IQR) length of ICU stay [2 (1–8) vs. 4 (2–10), respectively for PaCO_2_ ≥ 45 mmHg and < 45 mmHg; *p* = 0.101] and hospital stay [19 (9–30) vs. 21 (12–37), respectively for PaCO_2_ ≥ 45 mmHg and < 45 mmHg; *p* = 0.165] were not affected by baseline PaCO_2_ levels.

### Mortality

**Fig. 2:**
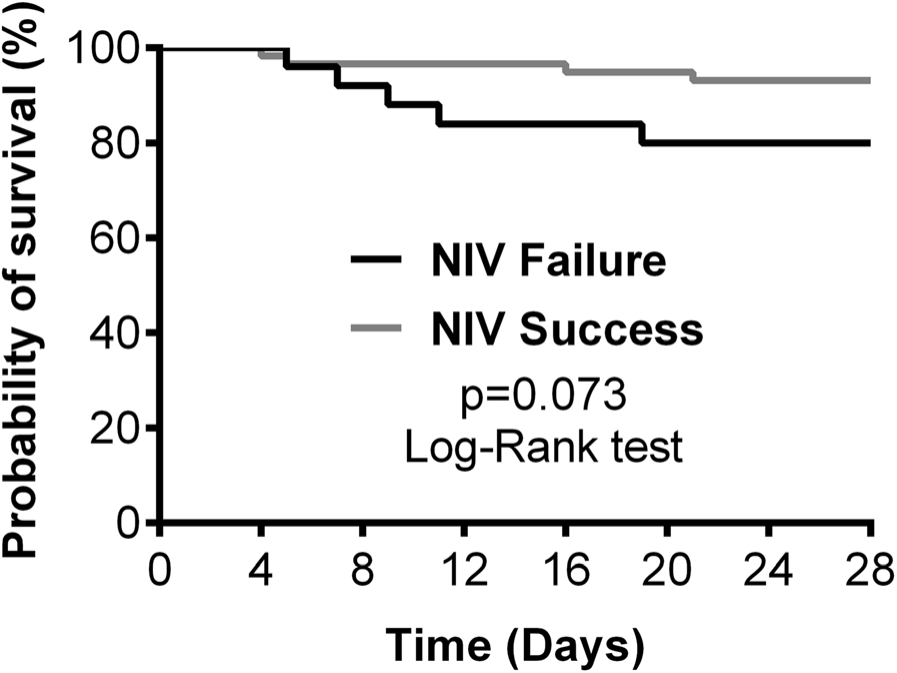
Kaplan-Meier curve for 28-day survival. NIV = noninvasive ventilation

In-hospital mortality rate was higher in the NIV failure patients compared to the NIV success patients [10/26 (38.5 %) vs. 7/59 (11.9 %), respectively for NIV failure and NIV success groups; *p* = 0.008] (Table 3). NIV failure was associated with an increased risk of in-hospital death (OR 4.64, 95 % CI 1.52 to 14.18; *p* = 0.007) while mortality at day 28 [5/26 (19.2 %) vs. 4/59 (6.8 %), respectively for NIV failure and NIV success groups; *p* = 0.124] did not differ between NIV failure and success groups (Table 3 and Figure 2).

In-hospital mortality [3/28 patients (10.7 %) vs. 14/57 patients (24.6 %), respectively for PaCO_2_ ≥ 45 mmHg and < 45 mmHg; *p* = 0.160] and 28-day mortality [3/28 patients (10.7 %) vs. 6/57 patients (10.5 %), respectively for PaCO_2_ ≥ 45 mmHg and < 45 mmHg, *p* = 1.000] did not differ between patients with baseline PaCO_2_ ≥ 45 mmHg or < 45 mmHg.

### Predictors of NIV failure

From the initial model containing 10 predictors, the backward elimination procedure yielded a reduced model containing age (OR 0.96, 95 % CI 0.93 to 0.99; *p* = 0.007) and APACHE II score (OR 1.13, 95 % CI 1.02 to 1.25; *p* = 0.018) (Table 4). Interaction between age and APACHE II score was not significant (*p* = 0.11).

**Table 4:** Logistic regression analysis addressing the main risk factors for noninvasive positive pressure ventilation failure

	Univariate analysis			Multivariate analysis		
Risk factors (predictors)	OR	95 % CI	*P* value	OR	95 % CI	*P* value
Age (years)	0.97	0.94–0.99	0.015	0.96	0.93–0.99	0.007
Male gender	2.24	0.86–5.83	0.100			
Mean arterial pressure (mmHg)	0.98	0.95–1.00	0.037			
Heart rate (bpm)	1.02	1.00–1.05	0.037			
APACHE II score	1.10	1.00–1.21	0.039	1.13	1.02–1.25	0.018
Arterial pH	0.95	0.89–1.01	0.116			
Bicarbonate (mmol/L)	0.87	0.78–0.97	0.009			
Arterial lactate (mg/dL)	1.05	0.99–1.10	0.081			
Transplantation	10.50	2.00–54.95	0.005			
Acute respiratory distress syndrome	2.57	0.68–9.80	0.167			

*OR* Odds ratio, *CI* Confidence interval, *APACHE* II Acute physiology and chronic health evaluation II (The score can range from 0 to 71, with higher scores indicating more severe illness)

## Discussion

This study showed a success rate of approximately 70 % of noninvasive ventilation in a general ICU population with acute hypoxemic respiratory failure. The initial PaCO_2_ levels (<45 mmHg or ≥ 45 mmHg) was not related to NIV failure/success. Patients who failed on NIV and received invasive mechanical ventilation were sicker, comparatively younger, had higher ICU and hospital length of stay and had higher in-hospital mortality rate. The multivariate logistic regression analysis showed that APACHE II score was an independent predictor of NIV failure, suggesting that sicker patients should be carefully monitored during the NIV trial regarding heart rate, mean arterial blood pressure and arterial lactate levels besides monitoring SpO_2_, PaO_2_, PaCO_2_, pH, respiratory rate and tidal volume for early prediction of NIV failure.

The main evidence-based clinical indications for NIV use in the critical care setting are exacerbations of chronic obstructive pulmonary disease [3, 4] and acute cardiogenic pulmonary edema [5–8]. Nevertheless, advances in NIV ventilators, development of more comfortable interfaces, improvement in patients monitoring and care during NIV delivery and staff training have contributed to the dissemination of NIV application in patients with ARF of different etiologies [19–21] and increased NIV use [20].

The success rate of NIV in critically ill patients can vary widely [1–11]. The main factors associated with success or failure were the etiology of respiratory insufficiency and the presence of dysfunction of other organs besides the lungs [2]. The reported success of NIV in hypoxemic respiratory failure is around 50 % while in hypercapnic respiratory failure it is around 75 % [2]. In our study population, 67 % (57/85) of patients had PaCO2 < 45 mmHg at baseline and the main reason for NIV start was desaturation while receiving supplementary oxygen through a Venturi mask or oxygen bag. The success of NIV in this group was approximately 67 % (38/57 patients), which shows that in everyday clinical practice, NIV should be attempted in the hypoxemic respiratory failure with two thirds of success without major complications when observing the use of an appropriate interface and NIV ventilator.

In our study, acute respiratory failure caused by community-acquired pneumonia was the main reason for NIV use. Intubation was avoided in 67 % (20/30) of the patients. Our results are in accordance to a recent report on the use of NIV in severe community-acquired pneumonia with acute respiratory failure that observed NIV success in 95 out of 127 (75 %) patients, suggesting that NIV should be a good option for patients with acute respiratory failure secondary to a community-acquired pneumonia [22].

Results in the medical literature suggest that NIV use in ARDS patients must be attempted with caution, due to high need of intubation and mortality rates associated with failure in these patients, especially in the more severe ones [23]. Recently, the use of NIV for acute hypoxemic failure was assessed in 82 ARDS and 31 non-ARDS patients over a 3-year period in an prospective cohort study [24]. Intubation rate was significantly higher in ARDS in comparison to non-ARDS patients (61 % vs. 35 %, *p* = 0.015) and varied according to the severity of disease: 31 % in mild, 62 % in moderate, and 84 % in severe ARDS (*p* = 0.0016) [24]. NIV failure was lower among moderate ARDS patients having a PaO_2_/FiO_2_ > 150 mmHg (45 % vs. 74 %, *p* = 0.04) [24].

Antonelli and colleagues showed in a randomized multicenter study that NIV was able to enhance oxygenation and avoid intubation in 54 % of ARDS patients [25]. Avoidance of intubation resulted in reduction of ventilator-associated pneumonia, ICU length of stay and mortality [25]. In our study, only 10 of our patients had the diagnosis of ARDS with a NIV success rate of 50 %. The median (IQR) length of ICU stay was 3.0 (3.0–5.0) days for ARDS patients treated successfully with NIV and 18.0 (10.0–34.0) days for those who required invasive mechanical ventilation (*p* = 0.008) although the in-hospital mortality did not differ between ARDS patients who failed on NIV in comparison to NIV success patients [3/5 (60 %) vs. 0/5 (0 %), *p* = 0.167]. According to these findings, instead of caution or contraindication of NIV use in ARDS patients, we suggest that a monitored ICU NIV trial should be considered in the ARDS patients due to the low mortality rates when NIV is successfully delivered [26]. However, in patients who failed the NIV trial, prompt intubation and invasive mechanical ventilation must be provided due to related high mortality rates in this population [26].

We found a higher prevalence of transplanted patients in the NIV failure group than in the NIV success group. A significant reduction in intubation rate and ICU length of stay using NIV for respiratory failure in recipients of solid organ transplantation have been reported [27–29]. Contrary to these findings, we observed a higher incidence of NIV failure in transplanted patients [7/9 (77.7 %)]. In our study, transplanted patients were comparatively younger than non-transplanted patients (45 ± 15 vs. 77 ± 13 years, respectively, *p* < 0.001). The younger age and higher failure rate in the transplanted patients may have contributed to the finding that comparatively younger age (67 ± 21 vs. 77 ± 14) was an independent predictor of NIV failure in our study.

In the present study, in-hospital mortality rate was higher in the NIV failure patients compared to the NIV success patients. Recently, Schnell and colleagues analyzed 1232 patients that received NIV out of 3163 (39 %) critically ill patients from a multicenter database [30]. First-line NIV was associated with better 60-day survival and fewer ICU-acquired infections compared to first line intubation in patients with acute-on-chronic respiratory failure [30].

Furthermore, it has been demonstrated that critically ill patients who required endotracheal intubation and invasive mechanical ventilation following a noninvasive ventilation exhibited a higher mortality rate than patients who were directly intubated [31–33]. Conversely, due to the increased risk of death attributed to NIV failure, a short period of NIV trial in hypoxemic respiratory patients has been proposed [34]. Nevertheless, the duration of the test and what specific population of hypoxemic patients this test should be applied in, is not well established in the literature. While a short period of a NIV trial may not be enough to allow the effects of NIV to be detectable, long periods on NIV may be associated with delayed initiation of mechanical ventilation and, therefore, to worst outcomes [31]. Therefore, a well-designed prospective controlled trial comparing a short well-monitored NIV trial to first line invasive mechanical ventilation in hypoxemic respiratory failure patients (excluding patients with absolute contra-indication or urgent need of intubation) is still needed.

Our study has limitations. This was an observational, prospective, single center study carried out in a general medical-surgical ICU for a strict period of three months and it included a small number of patients. Although our ICU has a protocol for the management of noninvasive ventilation, the identification of NIV failure and the indication for endotracheal intubation was based on the judgment of the attending physician. This variability in the day-by-day ICU medical care decisions is part of our real world and should be considered in mechanical ventilation studies. Finally, patients were ventilated with a full-face mask coupled to a BIPAP Vision® (ventilator specially designed for NIV delivery) that limits the interpretation of our results only to these settings.

## Conclusion

In our prospective cohort study, NIV failure in patients with acute respiratory failure was associated with increased in-hospital mortality, ICU and hospital stay and was not affected by baseline PaCO_2_ levels. Patients that failed were comparatively younger and had higher APACHE II score, suggesting the need for a careful selection of patients that might benefit from NIV and the need for a close monitoring in the more severe patients during NIV.

## Abbreviations

NIV: Noninvasive positive pressure ventilation
ARF: Acute respiratory failure
ICU: Intensive care unit
BIPAP: Bilevel positive airway pressure
CPAP: Continuous positive airway pressure
APACHE II: Acute physiology and chronic health evaluation II
PEEP: Positive end-expiratory pressure
SpO_2_: Peripheral oxygen saturation
PaO_2_: Arterial oxygen partial pressure
PaCO_2_: Arterial carbon dioxide partial pressure
FiO_2_: Fraction of inspired oxygen
PaO_2_/FiO_2_: Ratio of the arterial oxygen partial pressure to the fraction of inspired oxygen
ALI: Acute lung injury
ARDS: Acute respiratory distress syndrome
COPD: Chronic obstructive pulmonary disease
